# Synchronization in Interpersonal Speech

**DOI:** 10.3389/frobt.2019.00116

**Published:** 2019-11-08

**Authors:** Shahin Amiriparian, Jing Han, Maximilian Schmitt, Alice Baird, Adria Mallol-Ragolta, Manuel Milling, Maurice Gerczuk, Björn Schuller

**Affiliations:** ^1^ZD.B Chair of Embedded Intelligence for Health Care and Wellbeing, University of Augsburg, Augsburg, Germany; ^2^Group on Language, Audio & Music, Imperial College London, London, United Kingdom

**Keywords:** speech synchronization, human-human interaction, computational paralinguistics, machine learning, speech processing, autoencoders

## Abstract

During both positive and negative dyadic exchanges, individuals will often unconsciously imitate their partner. A substantial amount of research has been made on this phenomenon, and such studies have shown that synchronization between communication partners can improve interpersonal relationships. Automatic computational approaches for recognizing synchrony are still in their infancy. In this study, we extend on previous work in which we applied a novel method utilizing hand-crafted low-level acoustic descriptors and autoencoders (AEs) to analyse synchrony in the speech domain. For this purpose, a database consisting of 394 in-the-wild speakers from six different cultures, is used. For each speaker in the dyadic exchange, two AEs are implemented. Post the training phase, the acoustic features for one of the speakers is tested using the AE trained on their dyadic partner. In this same way, we also explore the benefits that deep representations from audio may have, implementing the state-of-the-art Deep Spectrum toolkit. For all speakers at varied time-points during their interaction, the calculation of reconstruction error from the AE trained on their respective dyadic partner is made. The results obtained from this acoustic analysis are then compared with the linguistic experiments based on word counts and word embeddings generated by our *word2vec* approach. The results demonstrate that there is a degree of synchrony during all interactions. We also find that, this degree varies across the 6 cultures found in the investigated database. These findings are further substantiated through the use of 4,096 dimensional Deep Spectrum features.

## 1. Introduction

It has been shown that during a dyadic human-human interaction, companions will often synchronize their communication style with their partner. This synchrony happens not only on a linguistic level, e.g., syntactic alignment (Gries, 2005; Dale and Spivey, 2006; Branigan et al., 2010), but also occurs across modes, with partners shifting their posture (Scheflen, 1964), facial expression (Blairy et al., 1999), as well as verbal cues (Chartrand and Bargh, 1999)—a topic which has been an area of interest across fields, including psychology (Likowski et al., 2012) and neuroscience (Seibt et al., 2015; Rymarczyk et al., 2018).

An alteration in the rapport between partners is one outcome in relation to synchronous behaviors, and can be described as an interpersonal aspect of a given dyadic exchange in which both partners are experiencing positivity (Tickle-Degnen and Rosenthal, 1990). From early-research in the field of psychology an increase in rapport was found from interactions in which body posture synchrony had occurred (LaFrance, 1979). However, due to the intrinsic complexity of human behavior, the measurement of interaction synchrony as an indicator of rapport has posed a substantial challenge for researchers (Bernieri et al., 1994). Nevertheless, in social psychological research a non-invasive measurement of interpersonal synchrony, which can be performed without the knowledge of participants, shows great potential for the analysis of human interaction (Bernieri et al., 1994).

Pickering and Garrod presented a mechanistic model of language processing during a dialogue (Pickering and Garrod, 2004). Their interactive alignment account describes how interlocutors automatically synchronize their linguistic representations on multiple levels, from syntax to semantics and phonetics. They argue that alignment on one level also increases alignment on other levels through mechanisms like *routinization* (i.e., the establishment of semi-fixed expressions encoding specific meanings). In recent years, approaches testing mimicry (synchrony) as a tool to enhance rapport have been popular in the field of Human Robot Interaction (HRI) (Riek et al., 2010; Li and Hashimoto, 2011). Valdesolo et al. analyzed the influence of synchrony on individuals who pursue joint goals (Valdesolo et al., 2010). The authors demonstrated that synchrony in body motions can enhance individuals' perceptual sensitivity to the movements of other persons and therefore can increase their success in a following cooperative task which requires the ability to respond appropriately to a partner's movement (Valdesolo et al., 2010). Furthermore, it was discussed that success in achieving common goals is motivated by the enhanced sense of collective spirit, and that synchrony could also predict cooperative ability (Valdesolo et al., 2010).

Previously studies in the area of automatic synchrony detection, have come largely from the vision domain (Michelet et al., 2012), some of which evaluating behaviors such as rate of head nods, and smiling (Sun et al., 2011a; Bilakhia et al., 2013). For this study, we focus on the acoustic signal, as it has been shown that aside from body-language, partners will additionally shift their speech style toward that of their partner (Giles, 1973; Giles et al., 1987).

Although there are similar previous works on this topic (Brdiczka et al., 2005; Burgoon and Hubbard, 2005), we have first proposed an acoustic-based approach to evaluate individual communication styles for the phenomenon of dyadic synchrony across a broad group of cultures (Han et al., 2018). First, we attempt a brute-force conventional approach in which we extract low-level descriptors (LLDs) such as log-energy, and pitch, to measure similarities in the speech turns, resulting in limited success (Han et al., 2018). To explore a state-of-the-art machine learning approach for this task, an autoencoder-based framework is implemented. The framework consists of two autoencoders (AEs), in which each is trained on the speech of one of the communication partners, subject A and B, respectively. On training completion, the data subsets are then switched, and fed to the opposing AE. In choosing this approach, we hypothesize that when a subject is behaving in a more synchronous manner, the reconstruction error of the features from the AE trained on their communication partner should decrease over time. Compared to other state-of-the-art computational approaches for unsupervised learning, e.g., Generative Adversarial Networks, AEs are relatively easy to train and chose hyperparameters for.

In the following section, the related work is summarized both from a sociological and a technical perspective. We then describe our multicultural dataset and the extracted acoustic and Deep Spectrum features used in our research. In section 4, we analyse the behavioral similarities of dyads and explain the experimental settings and discuss about our findings. Afterwards, in section 5, we analyse the linguistic behavior and compare the results to the ones obtained from our acoustic approach, before concluding the paper in section 6.

## 2. Related Work

Synchronous behavior (often referred to as mimicry), can play an important role as a mechanism of *emotional contagion* (Hatfield et al., 1993) i.e., the phenomenon an individual's emotional response to activate a similar emotion in their partner., and is either emotion- or motor-based (Hess and Fischer, 2013). Emotional synchrony is the change in affective states such as *happiness* or *anger*, and the motor-based synchrony would refer to physical changes, e.g., facial expression or position of the hands, although there is also literature indicating that vocal expression is often an unconscious motor act (McGettigan, 2015). Of the two, motor-based synchrony is a more effectively tracked aspect, as there is an object component which can be classified by a human observer, subsequently showing improved accuracy for automatic approaches such as body posture recognition (Hu et al., 2016).

Toward the end of the 1970s, the Facial Action Coding System' (Ekman and Friesen, 1978) based on so-called *facial action units* (FAUs), descriptors of 44 facial activations, was first proposed. Since this time FAUs have been utilized for an array of computational tasks (Kaiser and Wehrle, 1992; Tian et al., 2001; Jaiswal and Valstar, 2016). When combining active FAUs various facial expressions can be constructed, with a strong relationship between typical FAU combinations, e.g., frowning, or smiling, and an individual's affective state (Ekman and Friesen, 2003). These combinations have shown to be independent from culture (Ekman and Friesen, 2003), and can be robustly extracted utilizing state-of-the-art toolkits such as the well-known OpenFace (Baltrušaitis et al., 2016).

In general partners will likely show synchrony of traits such as gestures and posture, from their partner, nearer to the end of a conversation (Chartrand and Bargh, 1999; Delaherche et al., 2012). Motor-based synchrony can be applied as a persuasive tool during human-to-human exchange, specifically when including the mimicry of the partners spoken opinion (Hess and Fischer, 2013). From both the auditory and visual channels, humans are vulnerable to this behavior (Parrill and Kimbara, 2006). To this end, although there has been evidence of communication partners synchronizing when they do not agree, there is more prevalent factors of synchrony when partners discuss a common topic of which they hold a similar opinion (Sun et al., 2011a).

From a computational point of view, automatic detection approaches for motor-based synchronous behavior are varied. A time-based regression model which utilized long short-term memory (LSTM) recurrent neural networks (RNNs) was proposed as a prediction method for audio-visual features of *chat* partners (Bilakhia et al., 2013). In Bilakhia et al. (2013), the authors utilized *Mel-frequency cepstral coefficients* (MFCCs) as acoustic features and *facial landmarks* as visual features. They then trained an ensemble of models to predict the features of one chat partner based on the features of their dyadic partner in order to solve the binary classification task of *mimicry* or *non-mimicry*. The model in which the lowest reconstruction error was provided gave the class. In contrast to their work, our approach is unsupervised, i.e., the models are not trained to predict a ground truth occurrence of mimicry.

In general, emotion-based synchrony has not been extensively researched, and has shown to be highly dependent on social context, with individuals not synchronizing at all if they are not in favor with one another (Hess and Fischer, 2014). As well as having a positive outcome on negotiations (Swaab et al., 2011), a similar observation for the favored partner was found within linguistic information (Scissors et al., 2008). In a text-based interaction individuals were found to repeat the style of their partner over time, particularly in scenarios where trust was already established. In this same way, rapport during interactions was found to develop more highly between partners over time when repeating the counterpart's behaviors (LaFrance, 1979).

## 3. Dataset and Features

To validate the proposed approaches, we use the SEWA corpus of audio-visual interaction in-the-wild (Kossaifi et al., 2019)^1^. A database which has in the past been used as the official benchmark database for the 2017 and 2018 Audio-Visual Emotion Challenges (AVEC) (Ringeval et al., 2017, 2018). Extracting both hand-crafted acoustic features and deep representations of the audio signal on the frame-level of all sessions. We decided to extract both acoustic and Deep Spectrum features, due to their previous performance and proven ability in capturing characteristics of speech (Schuller et al., 2013; Amiriparian et al., 2016, 2018; Eyben, 2016). Both feature sets are different in their nature; ComParE is a hand-crafted, expert-designed feature set which can cover time-dependent frame-level information for the input signals, and Deep Spectrum is based on the spectrograms of audio signals, focusing mostly on the time-frequency properties of the speech.

### 3.1. The SEWA Video Chat Dataset

**Figure 1:**
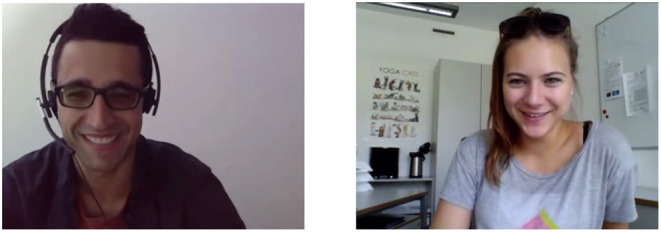
Screenshots taken from sample video chats in the SEWA corpus (German culture).

The SEWA database includes audio-visual recordings of 197 dyadic conversations (including 201 male and 197 female subjects), from individuals of six differing cultures (Chinese, Hungarian, German, British, Serbian, and Greek). A summary of the SEWA database is given in Table 1, including number and total duration of conversation for each culture. An example conversation is shown in Figure 1 and during such conversations, subjects discuss with each other their view of a 90 s advertisement of a (water) tap that they have just been shown via the web platform.

**Table 1:** SEWA corpus: Quantity of conversations and subjects, as well as total duration given in minutes for each culture.

**Index**	**Culture**	**# Conversations**	**# Subjects**	**Total duration**
C1	Chinese	35	70	101
C2	Hungarian	33	66	67
C3	German	32	64	89
C4	British	33	66	94
C5	Serbian	36	72	98
C6	Greek	28	56	81
Sum		197	394	530

The subjects were “in-the-wild” and using a personal computer, with recordings captured from either their home or office. The chat partners were already acquainted with one another before the chat (either family, friends, or colleagues), and included varied gender pairings (female-male, female-female, male-male), which were balanced across all sessions. Subject were aged between 18 and 60, and communication was held in the native language of the partners, with no specified limitation on what to discuss about the advertisement. From post analysis, it was found that the conversations in the SEWA Dataset contain a variety of emotional states, as well as instances of both agreement/disagreement, and additionally positive/negative rapport (Ringeval et al., 2017, 2018; Kossaifi et al., 2019).

### 3.2. Acoustic Features

The ComParE feature set of acoustic features (Eyben, 2016) is used for our first approach. For each audio recording, acoustic low-level descriptors are extracted using the openSMILE toolkit (Eyben et al., 2013) at a step size of 10 ms. ComParE LLDs are extracted at frame-level. *Functionals* defined in the feature set are not applied in this work, as the time-dependent frame-level information is of most interest. Extracted with a window size of 20 to 60 ms length, there are 65 LLDs in the ComParE feature set and these have been summarized in Table 2. Feature vectors of size 130 for each 10 ms step are given by calculating the first order derivative (deltas).

**Table 2:** Interspeech 2013 Computational Paralinguistics Challenge feature set.

**4 energy related LLD**	**Group**
Loudness	Prosodic
Modulation loudness	Prosodic
RMS energy, zero-crossing rate	Prosodic
**55 spectral related LLD**	**Group**
RASTA auditory bands 1–26	Spectral
MFCC 1–14	Cepstral
Spectral energy 250-650 Hz, 1–4 kHz	Spectral
Spectral roll-off pt. .25, .50, .75, .90	Spectral
Spectral flux, entropy, variance	Spectral
Spectral skewness and kurtosis	Spectral
Spectral slope	Spectral
Spectral harmonicity	Spectral
Spectral sharpness (auditory)	Spectral
Spectral centroid (linear)	Spectral
**6 voicing related LLD**	**Group**
*F*_0_ via SHS	Prosodic
Probability of voicing	Voice quality
Jitter (local and delta)	Voice quality
Shimmer	Voice quality
Log harmonics-to-noise ratio	Voice quality

*An overview of the 65 acoustic low-level descriptors (LLDs). SHS, Sub-Harmonic Summation*.

### 3.3. Deep Spectrum Features

In addition to the acoustic features (cf. section 3.2), we apply the feature extraction Deep Spectrum toolkit^2^ to extract deep representations from the audio signals using pre-trained convolutional neural networks (CNNs) (Amiriparian et al., 2017c). First, audio signals are transformed into Mel-spectrogram plots using a Hanning window of width 500 ms and an overlap 10 ms. From these, 128 Mel-frequency bands are then computed. Afterwards, the generated spectrograms are forwarded through VGG16 (Simonyan and Zisserman, 2014), a pre-trained CNN, and the activations of the penultimate fully connected layer (*fc7*) of the network are extracted, resulting in a 4,096 dimensional Deep Spectrum feature vector. These features can be considered as being a high-level representation of the Mel-spectrograms (Amiriparian et al., 2017c), and have shown to be highly effective in various speech and audio analysis tasks (Amiriparian et al., 2017a,c, 2018, 2019; Baird et al., 2017; Ringeval et al., 2018).

## 4. Behavior Similarity Tendency Analysis With Autoencoder

In order to investigate the temporal-based patterns, as well as interpersonal sentiment which may occur in speech, we first need to get machine readable representations from the speech signals of each individual (cf. section 3.2 and 3.3) and then use these features for our machine learning experiments (cf.section 4.1). Based on the experimental results (cf. section 4.2), we then analyse the behavior similarities in various cultures.

To minimize the variance between recording environments the acoustic features (130 frame level) are first standardized (zero mean and unit standard deviation) across the same recordings. We have neither standardized nor normalized the Deep Spectrum features, since we found during our preliminary evaluation that this negatively impacts autoencoder performance. Before beginning to train the AE (cf.section 4.1), the feature sequences are first segmented based on the transcriptions which are also included in the SEWA database. The feature sequences of each recording are then split in two sub-sequences, with each having the features of only one of the subjects.

We then use a machine learning framework based on autoencoders for investigating the effect of synchrony found in the feature sequences. Autoencoders are a special type of neural network architecture trained in an unsupervised manner to find a compact, information rich representation of the input data from which this input can be reconstructed (Vincent et al., 2008). Further, the reconstruction error that is made by a trained autoencoder on unseen test data can give an indication on how similar this data is to the training domain: In the context of audio analysis, this has for example been used for automatic acoustic novelty detection (Marchi et al., 2015), the intuition being that audio events that are foreign to the training data will be harder to accurately reconstruct for the autoencoder. For our experiments, the training domain of each autoencoder are the feature sequences of one speaker while the sequences of the speaker's partner are used for evaluation. In our approach, AEs use the features extracted at each frame as independent instances, without considering the evolution of features over time. For each individual dyadic interaction in the dataset, we proceed as follows: Features of one subject are applied frame-wise to train the first AE, with the features of the other used frame-wise for testing. Although training the AEs and reconstructing the features using each frame as an independent instance, we preserve the order of the test frames in order to generate the reconstructed sequence of features. Then, the root-mean-squared errors (RMSEs) are calculated between the reconstructed and actual features as a means of evaluating the extent to which the RMSE varies over time. For each conversation, we end with two AEs trained on the two subjects involved, with two one-dimensional RMSE sequences, whose slopes can be measured by computing their first derivatives and later averaged for further analysis.

### 4.1. Experimental Settings

**Figure 2:**
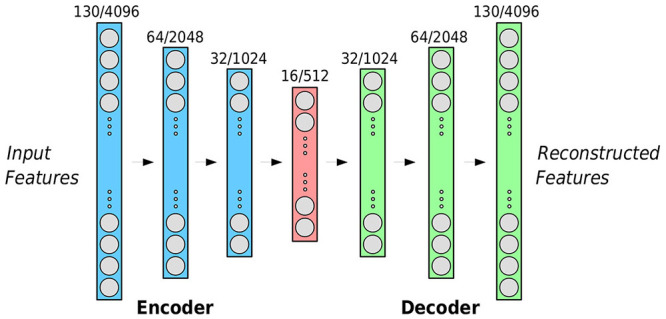
The autoencoder framework implemented for our study. The error between the input features (left) and the reconstructed features (right) is minimized for each subject using the RMSE. The given number of neurons in each layer (indicated above the neurons) refers to the compare /deep spectrum features, respectively.

For the AEs, we made use of a common bottleneck architecture: The input layer of the encoder and the output layer of the decoder match the size of the feature vectors whilst the size of neurons on the hidden layers is halved (doubled) for each layer in the encoder (decoder). As shown in Figure 2, the AE framework that has been constructed consists of a 3-layer encoder with a 3-layer decoder. During the initial experiments, nodes in each layer were selected as follows: 130–64–32–12–32–64–130, with the dimensions of the output matching that of the input low-level audio descriptors. For the Deep Spectrum features, we use a larger number of neurons on each layer: 4,096–2,048–1,024–512–1,024–2,048–4,096. We train all AEs with a batch size of 256 for 512 epochs minimizing the mean squared reconstruction error using the Adagrad (Duchi et al., 2011) optimizer with a learning rate of 0.01.

When the temporal reconstruction errors had been generated for each of the test subjects, the sequence is then utilized for a linear regression task, assuming that the learnt slope will indicate a behavior pattern change. In other words, when there is a negative slope, this may indicate that the dyadic partners are becoming more similar. Counter to this if there is a positive slope, it would indicate that the partners are less synchronized. As well as this, we make the additional assumption that the overall amplitude of the slope will denote the level of synchrony as well.

Our approach for using the slope for synchrony analysis between dyads is mainly motivated by the works introduced in Sun et al. (2011b), Delaherche et al. (2012), and Bilakhia et al. (2013). In Delaherche et al. (2012), the authors state that the interactive alignment/synchrony can be observed in conversation from a variety of features such as intonation, intensity, and rhythm in speech. In addition, in Bilakhia et al. (2013), the authors applied MSE to measure the reconstruction error of an unseen example with a trained model to detect non-verbal vocal mimicry vs. non-mimicry categories. In particular, 6 MFCCs were adopted as audio features instead of pitch or energy, whilst in the present work, more hand-crafted features, as well as deep representations, are investigated. Moreover, in Sun et al. (2011b), the results have shown that a long-term increasing correlation is consistently obtained between two speakers in a discussion. Thus, though the term “slope” was not well-supported in any of previous work, these previous findings motivate this work to adopt the RMSE slope overall interaction to indicate progressive synchronization. Furthermore, in Table 3, it has been demonstrated that the slope tendencies have a negative correlation with the answer to the question if an individual feels of holding the same opinion with the partner, demonstrating that the detected synchronization tendency has a high correlation with their self-reported labels.

**Table 3:** Average slope of RMSE sequences of all subjects and the Pearson correlation coefficients of pairs in each culture (C1: Chinese, C2: Hungarian, C3: German, C4: British, C5: Serbian, and C6: Greek).

**Feature set**	**C1**	**C2**	**C3**	**C4**	**C5**	**C6**
**Acoustic features**						
*average slope*	−0.07	−0.11	−0.10	−0.07	−0.08	−0.12
*pcc of pairs*	−0.03	0.34	0.15	0.39	0.39	−0.26
**DEEP SPECTRUM features**						
*average slope*	−0.03	−0.05	−0.03	−0.02	−0.05	−0.07
*pcc of pairs*	0.03	0.16	0.18	0.09	0.13	−0.15

*The autoencoders were trained on both acoustic and DEEP SPECTRUM features. For all cultures the average slope shallower when using DEEP SPECTRUM features*.

### 4.2. Results and Discussion

The first culture from the SEWA dataset; C1 (Chinese) will be where we begin our discussion. This culture consists of 35 sessions, and the average RMSE sequence slope for all 70 subjects is −0.07, and −0.03 when using acoustic and Deep Spectrum features, respectively. Using both feature sets, which differ in their nature, we show that very low average RMSE can be achieved for the Chinese culture. This finding indicates a relatively high synchrony between Chinese dyadic partners.

**Figure 3:**
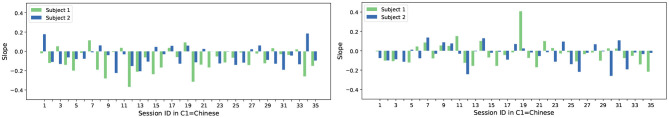
Slope of RMSE sequences of 70 Chinese subjects from 35 recordings. In each recording, there are two subjects as denoted with green and blue bars. The diagrams (from left to right) are generated based on the acoustic and Deep Spectrum features, respectively.

**Figure 4:**
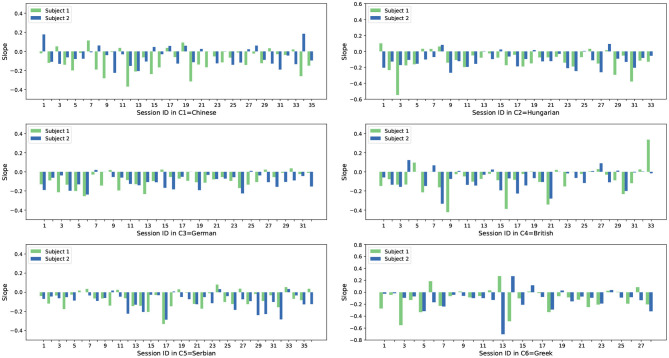
Slope of RMSE sequences of paired subjects from all recordings in all six cultures. The results are calculated based on the acoustic features.

**Figure 5:**
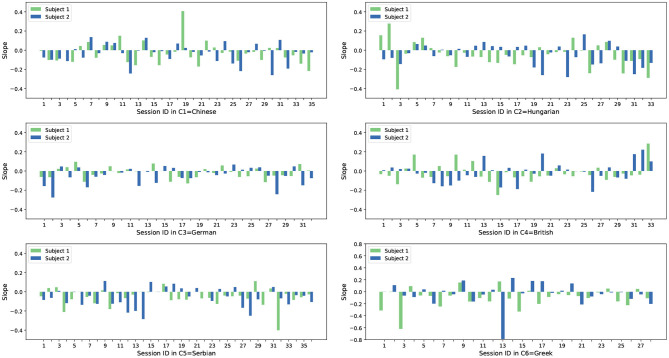
Slope of RMSE sequences of paired subjects from all recordings in all six cultures. The results are calculated based on the Deep Spectrum features.

From the analysis shown in Figure 3 it can be seen that most subject slopes for both feature sets (54 /70 for the acoustic features and 47 /70 for the Deep Spectrum features) are negative, with less being positive. With our previous assumption in mind, these results indicate that the acoustic LLD features and the Deep Spectrum features of these subjects have a smaller reconstruction error over time. As the AE is trained with the opposing subject from the same session a smaller reconstruction error should indicate higher synchrony between the communication partners. We also see a similar trend across other cultures in the dataset, however the ratios for negative / positive slope vary across cultures. Figures 4, 5 show the slope of RMSE for all subjects and all cultures obtained from both feature sets.

With these results in mind, the average slopes *s* were calculated for all cultures, as well as the Pearson correlation coefficients (PCCs). This was made with the intention of investigating cultural-based variation across the spontaneous in the wild conversations. For this analysis, results are summarized in Table 3. As mentioned a negative slope indicates a more synchronous speech-based relationship. The *average slope* is computed to demonstrate the overall tendency throughout all subjects in one specific culture, whilst the *pcc of pairs* is applied to indicate the tendency between two conversation partners given that specific culture.

From the correlation analysis shown in Table 3, it can be noticed that generally when observed as group pairings A/B, individuals across the six cultures show a tendency to synchronize. Given that *s* for each culture is always negative. The Greek culture (C6) shows the largest slope, i.e., lower synchrony between the Greek dyads, and the smallest slope is observed for both Chinese and British cultures.

As well as this, when looking only at the PCC, we can see an alternative culture variance. In the case of PCC, positive values indicate that the subjects of a culture converge to a similar place, either both behaving in synchrony or out of synchrony with one another. Conversely, a negative PCC would indicate that one subject is dominating the other. No correlation is seen in the C1 (Chinese) pairs for example, with a PCC of −0.03 and 0.03 when using acoustic and Deep Spectrum features which is close to 0. On the other side, a linear correlation is shown as either positive for the Hungarian (C2), German (C3), British (C4), and Serbian (C5) or negative for the Greek (C6) culture. Although out of the scope of our study, it would be of benefit to verify these findings based on literature across other fields, such as the anthropological linguistics domain and the field of conversation analysis (Stivers et al., 2009). We should also note that variances such as educational background, occupation, and health status of the individuals in the SEWA dataset may have some effect on the result, however, although the dataset providers did implement a control of aspects such as age and gender, variation between complex characteristics such as these would be difficult to avoid.

## 5. Linguistic Behavior Analysis and Similarity Patterns

Motor-based synchrony, e.g., raising an eyebrow, can be detected from visual mid-level features such as Facial Action Units (FAUs) (Surakka and Hietanen, 1998). Nonetheless, the detection of similarity in speech from raw features is challenging due to the variability of speech descriptors. To name a few, these descriptors are sensitive to the environment and the voice of the subject, which is influenced by factors such as age and gender, amongst others.

Besides the acoustic similarities, we should also investigate the behavioral synchronization shown in video chats from other modalities, including linguistic information. In this regard, rather than the conventional bag-of-words (BoW) approach, which represents a text as a sparse histogram vector, word embeddings are the current state-of-the-art (Kusner et al., 2015; Liu et al., 2015; Amiriparian et al., 2017b; Chung and Glass, 2018). With this technique, the sparse histogram vectors, with a dimensionality higher than ℝ^1×5000^, are transformed into a lower dimensionality vector, typically ℝ^1×300^, where each component in the vector space represents a concept. As a relevant property of word embeddings, the distance between this concept and words with similar meanings is lower than the distance between this concept and words with completely different meanings. The architecture of neural networks for word embeddings usually includes a single layer, which converts the BoW into the embedding vector. Currently, *word2vec*, introduced by Mikolov et al. (2013), is a popular technique to generate word embeddings, as it is trained on large text corpora, such as Wikipedia. This technique employs a specialized objective function, called“negative sampling.” One of the benefits of using such word embedding technique is that the representations generated from the words quantitatively capture several properties of the object they describe (Mikolov et al., 2013).

We base our analysis on the manual transcriptions of the video chats from the six different cultures included in the SEWA database (cf. section 3 for details). Word embeddings are extracted using pre-trained *word2vec* models available on the internet. While a word embedding model for the British culture trained on a Google News corpus is employed^3^, word embedding models for the Hungarian and German cultures trained on Wikipedia dumps are used^4^. For the other cultures, suitable word embedding pre-trained models are not currently available and, as a consequence, we exclude these cultures from our experiments with the *word2vec* approach. Furthermore, training our own word embedding models on the transcriptions of the SEWA database is discarded due to limitations on the available data. Word embedding models require large amounts of data to be trained, usually requiring more than a million running words.

In order to analyse the linguistic synchronization as the interaction progresses, we decide to split the chat sessions in two halves, the first and second half of each conversation. The measurement of similarities on a smaller scale, e.g., on utterance or speaker turn level, is not possible, as some particular speaker turns are quite long (more than 30 s). For every half of the interaction *word2vec* embeddings are extracted from both the speaker and their partner, and the cosine similarity between the word embeddings is computed. In addition to word embeddings, a simple evaluation of word usage is also made by counting how often the same words were used by the two subjects in each segment and normalizing the result by the number of words per segment. The averaged similarities of both scenarios in both halves of the interactions for all participants belonging to the same culture are calculated and summarized in Table 4.

**Table 4:** Evaluation of linguistic similarities between dyadic companions in the two halves of the video chat.

**Culture**	**Word usage similarity**		***word2vec*** **similarity**	
	**1^*st*^ half**	**2^*nd*^ half**	**1^*st*^ half**	**2^*nd*^ half**
C1 (Chinese)	0.710	0.880	—	—
C2 (Hungarian)	0.738	0.902	0.809	0.794
C3 (German)	1.063	1.128	0.301	0.327
C4 (British)	1.714	1.787	0.364	0.383
C5 (Serbian)	1.241	1.353	—	—
C6 (Greek)	0.849	1.125	—	—

*The linguistic information is analyzed using two different approaches: by computing word usage and by extracting word2vec embeddings from the transcripts included in the SEWA database*.

The results reported in Table 4 show that for all cultures the linguistic similarity increases during the video chat in regards to the word usage. For *word2vec* embeddings the increase is very subtle and in particular, for the Hungarian culture, we observe that the similarity slightly decreases. The very weak or even non-existent linguistic synchronization we measured with the *word2vec* approach could be explained by the nature of the rather complex features. It seems possible that a synchronization on such a high linguistic level takes even more time than the acoustic synchronization or the linguistic synchronization on the word level and could therefore not be measured in short conversations. This result leads us to assume that rapport and synchrony in the linguistic domain is manifested in the direct synchrony of terminology, rather than in synchrony of concepts and topics.

The differences of linguistic similarity across cultures is quite noticeable as the values of word usage similarity in the first half of the conversations range from 0.710 in the Chinese culture up to 1.714 in the British culture. In the *word2vec* approach the similarity values for the first half of the conversations range from 0.301 in the German culture up to 0.809 in the Hungarian culture. Reasons for this, as for the different changes of the similarity through the conversations, might lie in the respective languages of the different cultures or culture-specific behaviors during conversation.

## 6. Conclusion and Outlook

In this work, we have demonstrated that, an autoencoder-based framework has great potential to recognize the spontaneous and unconscious synchronization which occur during social interactions. We can see this evidence through the observation of the reconstruction error, when using the acoustic and Deep Spectrum features extracted from the speech of each dyadic companion.

From this work, we have also explored culturally dependent synchronization of vocal behavior in dyadic conversations. In section 4, we have analyzed the behavior similarities and ability of interpersonal chats to synchronize. It was found that both feature sets are suitable for this task. Most subjects slopes are negative when observing the feature sets (54 /70 for the acoustic features and 47 /70 for the Deep Spectrum features). From additional correlation analysis, it was found that individuals do tend to synchronize, however from this analysis, the cultural differences were more noticeable, e.g., C6 (Greek) and C1 (Chinese) show quite opposing average slopes (−0.07 and −0.03, respectively with Deep Spectrum features).

Furthermore, the results provided in Table 4 demonstrated that for all six cultures the linguistic similarity increases during the video chat.

Future work will focus on utilizing further unsupervised representation learning techniques, such as unsupervised feature learning with deep neural networks using the auDeep toolkit (Amiriparian et al., 2017b; Freitag et al., 2018), and feature quantization methods, such as *bag-of-audio-words* (Schmitt et al., 2016). Moreover, we are planing to exploit the linguistic domain through state-of-the-art *word2vec* embeddings (Mikolov et al., 2013). Given the findings in relation to cultures from the utilized dataset, it would also be of value to further explore this, possibly through a deeper analysis of non-verbal synchrony and the known occurrence of this during dyadic interactions (Tschacher et al., 2014). It is also of big interest to analyse the amount of alignment between speakers across different dyads. Finally, in addition to the slope of the reconstruction errors, we want to explore further evaluation strategies to measure the degree of synchrony between subjects (Delaherche et al., 2012).

## Data Availability Statement

The dataset analyzed for this study, SEWA, is a public dataset and can be found under the following link: https://db.sewaproject.eu/.

## Ethics Statement

For recording the SEWA dataset the local ethics board, the Imperial College Research Ethics Committee (ICREC), has approved the recording of the audio-visual database and the study of audio-visual behavior in the collected data. All subjects analyzed for the study described in this article have given their written informed consent to participate prior to recording. The two participants shown in Figure 1 have given their written informed consent to publish excerpts from their recordings in academic documents, articles, and presentations.

## Author Contributions

SA, JH, and MS conceptualized the study and ran the machine learning experiments. AB, AM-R, MM, and BS did literature analysis, manuscript preparation and editing. MG helped with running the experiments and testing the codes. All authors revised, developed, read, and approved the final manuscript.

### Conflict of Interest

The authors declare that the research was conducted in the absence of any commercial or financial relationships that could be construed as a potential conflict of interest.

## Footnotes

^1^https://sewaproject.eu/

^2^https://github.com/DeepSpectrum/DeepSpectrum

^3^https://github.com/3Top/word2vec-api

^4^https://github.com/Kyubyong/wordvectors

## References

[B1] Amiriparian S. Cummins N. Gerczuk M. Pugachevskiy S. Ottl S. Schuller B. (2019). “Are you playing a shooter again?!” deep representation learning for audio-based video game genre recognition. IEEE Trans. Games 11 10.1109/TG.2019.2894532

[B2] Amiriparian S. Cummins N. Ottl S. Gerczuk M. Schuller B. (2017a). Sentiment analysis using image-based deep spectrum features, in Proceedings of the 7th Biannual Conference on Affective Computing and Intelligent Interaction (ACII 2017) (San Antonio, TX), 26–29.

[B3] Amiriparian S. Freitag M. Cummins N. Schuller B. (2017b). Sequence to sequence autoencoders for unsupervised representation learning from audio, in Proceedings of the DCASE 2017 Workshop (Munich), 17–21.

[B4] Amiriparian S. Gerczuk M. Ottl S. Cummins N. Freitag M. Pugachevskiy S. (2017c). Snore sound classification using image-based deep spectrum features, in Proceedings of INTERSPEECH 18th Annual Conference of the International Speech Communication Association (Stockholm: ISCA), 3512–3516.

[B5] Amiriparian S. Gerczuk M. Ottl S. Cummins N. Pugachevskiy S. Schuller B. (2018). Bag-of-deep-features: Noise-robust deep feature representations for audio analysis, in Proceedings of the 31st International Joint Conference on Neural Networks (IJCNN) (Rio de Janeiro: IEEE), 2419–2425.

[B6] Amiriparian S. Pohjalainen J. Marchi E. Pugachevskiy S. Schuller B. (2016). Is deception emotional? An emotion-driven predictive approach, in Proceedings INTERSPEECH 2016, 17th Annual Conference of the International Speech Communication Association (San Francisco, CA: ISCA), 2011–2015.

[B7] Baird A. Amiriparian S. Cummins N. Alcorn A. M. Batliner A. Pugachevskiy S. (2017). Automatic classification of autistic child vocalisations: A novel database and results, in Proceedings of INTERSPEECH 2017, 18th Annual Conference of the International Speech Communication Association (Stockholm: ISCA), 849–853.

[B8] Baltrušaitis T. Robinson P. Morency L.-P. (2016). OpenFace: an open source facial behavior analysis toolkit, in Proceedings of the IEEE Winter Conference on Applications of Computer Vision (WACV) (Lake Placid, NY), 1–10.

[B9] Bernieri F. J. Davis J. M. Rosenthal R. Knee C. R. (1994). Interactional synchrony and rapport: measuring synchrony in displays devoid of sound and facial affect. Pers. Soc. Psychol. Bull. 20, 303–311. 10.1177/0146167294203008

[B10] Bilakhia S. Petridis S. Pantic M. (2013). Audiovisual detection of behavioural mimicry, in Proceedings Humaine Association Conference on Affective Computing and Intelligent Interaction (ACII) (Geneva), 123–128.

[B11] Blairy S. Herrera P. Hess U. (1999). Mimicry and the judgement of emotional facial expressions. J. Nonverbal Behav. 23, 5–41. 10.1023/A:1021370825283

[B12] Branigan H. P. Pickering M. J. Pearson J. McLean J. F. (2010). Linguistic alignment between people and computers. J. Pragmatics 42, 2355–2368. 10.1016/j.pragma.2009.12.012

[B13] Brdiczka O. Maisonnasse J. Reignier P. (2005). Automatic detection of interaction groups, in Proceedings of the 7th International Conference on Multimodal Interfaces, ICMI '05 (Trento), 32–36.

[B14] Burgoon J. K. Hubbard A. E. (2005). Cross-cultural and intercultural applications of expectancy violations theory and interaction adaptation theory, in Theorizing About Intercultural Communication, ed Gudykunst W. B. (Thousand Oaks, CA: Sage) 149–171.

[B15] Chartrand T. L. Bargh J. A. (1999). The chameleon effect: the perception–behavior link and social interaction. J. Pers. Soc. Psychol. 76, 893–910. 10.1037//0022-3514.76.6.893 10402679

[B16] Chung Y.-A. and Glass, J. (2018). Speech2vec: a sequence-to-sequence framework for learning word embeddings from speech. arXiv preprint arXiv:1803.08976.

[B17] Dale R. Spivey M. J. (2006). Unraveling the dyad: using recurrence analysis to explore patterns of syntactic coordination between children and caregivers in conversation. Lang. Learn. 56, 391–430. 10.1111/j.1467-9922.2006.00372.x

[B18] Delaherche E. Chetouani M. Mahdhaoui A. Saint-Georges C. Viaux S. Cohen D. (2012). Interpersonal synchrony: a survey of evaluation methods across disciplines. IEEE Trans. Affect. Comput. 3, 349–365. 10.1109/T-AFFC.2012.12

[B19] Duchi J. Hazan E. Singer Y. (2011). Adaptive subgradient methods for online learning and stochastic optimization. J. Mach. Learn. Res. 12, 2121–2159.

[B20] Ekman P. Friesen W. V. (1978). Facial Action Coding System. Consulting Psychologists Press. Available online at: https://books.google.fr/books?id=08l6wgEACAAJ

[B21] Ekman P. Friesen W. V. (2003). Unmasking the Face: A Guide to Recognizing Emotions From Facial Clues, 1 Edn Los Altos, CA: Ishk.

[B22] Eyben F. (2016). Real-Time Speech and Music Classification by Large Audio Feature Space Extraction, 1 Edn Basel: Springer.

[B23] Eyben F. Weninger F. Groß F. Schuller B. (2013). Recent developments in openSMILE, the Munich open-source multimedia feature extractor, in Proceedings the 21st ACM International Conference on Multimedia (ACMM) (Barcelona), 835–838.

[B24] Freitag M. Amiriparian S. Pugachevskiy S. Cummins N. Schuller B. (2018). audeep: Unsupervised learning of representations from audio with deep recurrent neural networks. J. Mach. Learn. Res. 18, 1–5.

[B25] Giles H. (1973). Accent mobility: a model and some data. Anthropol. Linguist. 15, 87–105.

[B26] Giles H. Mulac A. Bradac J. J. Johnson P. (1987). Speech accommodation theory: the first decade and beyond. Ann. Int. Commun. Assoc. 10, 13–48. 10.1080/23808985.1987.11678638

[B27] Gries S. T. (2005). Syntactic priming: a corpus-based approach. J. Psycholinguist. Res. 34, 365–399. 10.1007/s10936-005-6139-3 16142588

[B28] Han J. Schmitt M. Schuller B. W. (2018). You sound like your counterpart: Interpersonal speech analysis, in Proceedings of Speech and Computer - 20th International Conference, SPECOM (Leipzig), 188–197.

[B29] Hatfield E. Cacioppo J. T. Rapson R. L. (1993). Emotional contagion. Curr. Dir. Psychol. Sci. 2, 96–100. 10.1017/CBO9781139174138

[B30] Hess U. Fischer A. (2013). Emotional mimicry as social regulation. Pers. Soc. Psychol. Rev. 17, 142–157. 10.1177/1088868312472607 23348982

[B31] Hess U. Fischer A. (2014). Emotional mimicry: why and when we mimic emotions. Soc. Pers. Psychol. Compass 8, 45–57. 10.1111/spc3.12083

[B32] Hu F. Wang L. Wang S. Liu X. He G. (2016). A human body posture recognition algorithm based on bp neural network for wireless body area networks. China Commun. 13, 198–208. 10.1109/CC.2016.7563723

[B33] Jaiswal S. Valstar M. (2016). Deep learning the dynamic appearance and shape of facial action units, in Proceedings of 2016 IEEE Winter Conference on Applications of Computer Vision (WACV) (New York, NY: IEEE), 1–8.

[B34] Kaiser S. Wehrle T. (1992). Automated coding of facial behavior in human-computer interactions with facs. J. Nonverbal Behav. 16, 67–84. 10.1007/BF00990323

[B35] Kossaifi J. Walecki R. Panagakis Y. Shen J. Schmitt M. Ringeval F.. (2019). SEWA DB: a rich database for audio-visual emotion and sentiment research in the wild. CoRR, abs/1901.02839. 31581074 10.1109/TPAMI.2019.2944808

[B36] Kusner M. Sun Y. Kolkin N. Weinberger K. (2015). From word embeddings to document distances, in International Conference on Machine Learning (Lille), 957–966.

[B37] LaFrance M. (1979). Nonverbal synchrony and rapport: Analysis by the cross-lag panel technique. Soc. Psychol. Q. 42, 66–70. 10.2307/3033875

[B38] Li Y. Hashimoto M. (2011). Effect of emotional synchronization using facial expression recognition in human-robot communication, in Proceedings of 2011 IEEE International Conference on Robotics and Biomimetics (ROBIO) (Phuket), 2872–2877.

[B39] Likowski K. Muehlberger A. Gerdes A. Wieser M. Pauli P. Weyers P. (2012). Facial mimicry and the mirror neuron system: simultaneous acquisition of facial electromyography and functional magnetic resonance imaging. Front. Hum. Neurosci. 6:214. 10.3389/fnhum.2012.00214 22855675 PMC3405279

[B40] Liu Y. Liu Z. Chua T.-S. Sun M. (2015). Topical word embeddings, in Proceedings of Conference on Artificial Intelligence (AAAI).

[B41] Marchi E. Vesperini F. Eyben F. Squartini S. Schuller B. (2015). A novel approach for automatic acoustic novelty detection using a denoising autoencoder with bidirectional lstm neural networks, in 2015 IEEE International Conference on Acoustics, Speech and Signal Processing (ICASSP) (Brisbane: IEEE), 1996–2000.

[B42] McGettigan C. (2015). The social life of voices: studying the neural bases for the expression and perception of the self and others during spoken communication. Front. Hum. Neurosci. 9:129. 10.3389/fnhum.2015.00129 25852517 PMC4365687

[B43] Michelet S. Karp K. Delaherche E. Achard C. Chetouani M. (2012). Automatic imitation assessment in interaction, in Human Behavior Understanding, eds Salah A. A. Ruiz-del Solar J. Meriçli Ç. Oudeyer P. Y. (Berlin; Heidelberg: Springer), 161–173.

[B44] Mikolov T. Sutskever I. Chen K. Corrado G. S. Dean J. (2013). Distributed representations of words and phrases and their compositionality, in Proceedings of NIPS (Lake Tahoe, NV), 3111–3119.

[B45] Parrill F. Kimbara I. (2006). Seeing and hearing double: the influence of mimicry in speech and gesture on observers. J. Nonverbal Behav. 30:157 10.1007/s10919-006-0014-2

[B46] Pickering M. J. Garrod S. (2004). Toward a mechanistic psychology of dialogue. Behav. Brain Sci. 27, 169–190. 10.1017/S0140525X04000056 15595235

[B47] Riek L. D. Paul P. C. Robinson P. (2010). When my robot smiles at me:enabling human-robot rapport via real-time head gesture mimicry. J. Multimodal User Interfaces 3, 99–108. 10.1007/s12193-009-0028-2

[B48] Ringeval F. Schuller B. Valstar M. Cowie R. Kaya H. Schmitt M. (2018). Avec 2018 workshop and challenge: bipolar disorder and cross-cultural affect recognition, in Proceedings of the 2018 on Audio/Visual Emotion Challenge and Workshop (AVEC) (Seoul: ACM), 3–13.

[B49] Ringeval F. Schuller B. Valstar M. Gratch J. Cowie R. Scherer S. (2017). AVEC 2017: Real-life depression, and affect recognition workshop and challenge, in Proceedings of n Proceedings of the 2018 on Audio/Visual Emotion Challenge and Workshop (AVEC) (Mountain View, CA), 3–9.

[B50] Rymarczyk K. Zurawski L. Jankowiak-Siuda K. Szatkowska I. (2018). Neural correlates of facial mimicry: Simultaneous measurements of emg and bold responses during perception of dynamic compared to static facial expressions. Front. Psychol. 9:52. 10.3389/fpsyg.2018.00052 29467691 PMC5807922

[B51] Scheflen A. E. (1964). The significance of posture in communication systems. Psychiatry 27, 316–331. 10.1080/00332747.1964.11023403 14216879

[B52] Schmitt M. Ringeval F. Schuller B. (2016). At the border of acoustics and linguistics: Bag-of-Audio-Words for the recognition of emotions in speech, in Proceedings INTERSPEECH 2017, 17th Annual Conference of the International Speech Communication Association (San Francisco, CA), 495–499.

[B53] Schuller B. Steidl S. Batliner A. Vinciarelli A. Scherer K. Ringeval F. (2013). The INTERSPEECH 2013 computational paralinguistics challenge: social signals, conflict, emotion, autism, in Proceedings of INTERSPEECH (Lyon), 148–152.

[B54] Scissors L. E. Gill A. J. Gergle D. (2008). Linguistic mimicry and trust in text-based cmc, in Proceedings of the ACM Conference on Computer Supported Cooperative Work (San Diego, CA), 277–280.

[B55] Seibt B. Mühlberger A. Likowski K. Weyers P. (2015). Facial mimicry in its social setting. Front. Psychol. 6:1122. 10.3389/fpsyg.2015.01122 26321970 PMC4531238

[B56] Simonyan K. Zisserman A. (2014). Very deep convolutional networks for large-scale image recognition. CoRR, abs/1409.1556.

[B57] Stivers T. Enfield N. J. Brown P. Englert C. Hayashi M. Heinemann T.. (2009). Universals and cultural variation in turn-taking in conversation. Proc. Natl. Acad. Sci. U.S.A. 106, 10587–10592. 10.1073/pnas.0903616106 19553212 PMC2705608

[B58] Sun X. Nijholt A. Truong K. P. Pantic M. (2011a). Automatic visual mimicry expression analysis in interpersonal interaction, in Proceedings of IEEE Computer Society Conference on Computer Vision and Pattern Recognition Workshops (CVPRW) (Colorado Springs, CO), 40–46.

[B59] Sun X. Truong K. P. Pantic M. Nijholt A. (2011b). Towards visual and vocal mimicry recognition in human-human interactions, in 2011 IEEE International Conference on Systems, Man, and Cybernetics (Anchorage, AK: IEEE), 367–373.

[B60] Surakka V. Hietanen J. K. (1998). Facial and emotional reactions to duchenne and non-duchenne smiles. Int. J. Psychophysiol. 29, 23–33. 10.1016/S0167-8760(97)00088-3 9641245

[B61] Swaab R. I. Maddux W. W. Sinaceur M. (2011). Early words that work: when and how virtual linguistic mimicry facilitates negotiation outcomes. J. Exp. Soc. Psychol. 47, 616–621. 10.1016/j.jesp.2011.01.005

[B62] Tian Y.-I. Kanade T. Cohn J. F. (2001). Recognizing action units for facial expression analysis. IEEE Trans. Pattern Anal. Mach. Intellig. 23, 97–115. 10.1109/34.908962 25210210 PMC4157835

[B63] Tickle-Degnen L. Rosenthal R. (1990). The nature of rapport and its nonverbal correlates. Psychol. Inquiry 1, 285–293. 10.1207/s15327965pli0104_1

[B64] Tschacher W. Rees G. M. Ramseyer F. (2014). Nonverbal synchrony and affect in dyadic interactions. Front. Psychol. 5:1323. 10.3389/fpsyg.2014.01323 25505435 PMC4241744

[B65] Valdesolo P. Ouyang J. DeSteno D. (2010). The rhythm of joint action: synchrony promotes cooperative ability. J. Exp. Soc. Psychol. 46, 693–695. 10.1016/j.jesp.2010.03.004

[B66] Vincent P. Larochelle H. Bengio Y. Manzagol P.-A. (2008). Extracting and composing robust features with denoising autoencoders, in Proceedings of the 25th International Conference on Machine Learning (Helsinki: ACM), 1096–1103.

